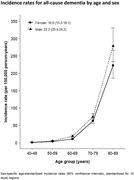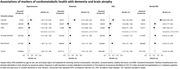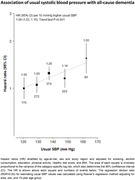# Cardiometabolic health and dementia risk: A prospective cohort study of 0.5 million adults in China

**DOI:** 10.1002/alz70860_098845

**Published:** 2025-12-23

**Authors:** Clara Bueno Lopez, Fiona Bragg, Andri Iona, Zhengming Chen, China Kadoorie Biobank

**Affiliations:** ^1^ University of Oxford, Oxford, Oxfordshire, United Kingdom

## Abstract

**Background:**

China faces one of the largest dementia‐associated economic and healthcare burdens globally, accounting for approximately one‐fourth of dementia cases worldwide. However, risk factors for dementia are poorly understood in the Chinese population. We investigate the relevance of markers of cardiometabolic health for risk of dementia.

**Method:**

China Kadoorie Biobank is a prospective cohort study involving 512,724 adults aged 30‐79 years, recruited in 2004‐2008 from 10 diverse regions. During ∼12 years’ follow‐up, 1,099 cases of dementia were recorded, including 173 vascular and 344 Alzheimer's dementia, through linked death registries and health insurance databases. Cox regression yield adjusted hazard ratios (HRs) for incident dementia associated with markers of cardiometabolic health.

**Result:**

At recruitment, 34.5% of participants had hypertension, 6.0% diabetes, 3.1% IHD and 1.8% stroke/transient ischaemic attack (TIA). By 2018, an additional 26,652 incident cases of hypertension, 23,747 of diabetes, 56,504 of IHD, and 64,744 of stroke/TIA were recorded. The incidence rate for dementia was 19.0 (95% CI: 17.9‐20.1) per 100,000 person‐years, higher at older ages, among men and in rural regions. There were positive associations of prior hypertension (HR 1.21 [95% CI 1.05‐1.39]), diabetes (1.36 [1.13‐1.65]), stroke/TIA (2.52 [2.14‐2.96]) and, less clearly, IHD (1.17 [0.97‐1.41]) with dementia, driven by notably strong associations with vascular dementia. Systolic blood pressure (SBP) showed a log‐linear positive trend with dementia risk, with 9% higher risk (1.09 [1.03‐1.15]) per 10 mmHg higher usual SBP.

**Conclusion:**

In this population with a high vascular disease burden, markers of cardiometabolic health are important risk factors for dementia.